# Sexual risk behaviour and its correlates among adolescents in Mozambique: results from a national school survey in 2015

**DOI:** 10.1080/17290376.2020.1858947

**Published:** 2021-02-28

**Authors:** Supa Pengpid, Karl Peltzer

**Affiliations:** a ASEAN Institute for Health Development, Mahidol University, Nakhon Pathom, Thailand; b Department of Research Administration and Development, University of Limpopo, Turfloop, South Africa; c Department of Psychology, University of the Free State, Bloemfontein, South Africa

**Keywords:** Sexual behaviour, adolescents, health risk behaviour, Mozambique

## Abstract

The study aimed to assess the prevalence and correlates of sexual risk behaviours among adolescents in Mozambique. In the cross-sectional ‘Global School-Based Health Survey (GSHS)’, 1918 students aged 11–18 years from Mozambique responded to a questionnaire in 2015. More than half (57.4%) of the students ever had sex, 68.4% among boys and 45.8% among girls. Among students who ever had sex, 41.5% had early sexual debut (<14 years), 57.9% had multiple sexual partners, 25.0% had not used a condom and 42.0% had not used birth control at last sex, and 59.4% engaged in multiple sexual risk behaviour. In adjusted logistic regression analysis, alcohol use, school truancy, older age and male sex were associated with multiple sexual risk behaviours. A large number of adolescents in Mozambique reported sexual risk behaviours, emphasising the need for interventions.

## Introduction

In the adolescent period sexual activity and sexual risk behaviours, such as unprotected sex, may be initiated (Bearinger, Sieving, Ferguson, & Sharma, 2007). In studies among adolesents in African countries, various sexual risk behaviours have been identified (Doyle, Mavedzenge, Plummer, & Ross, 2012). The prevalence of early sexual debut (<15 years) was 22% among girls and 17% among boys (15–19 years) in 2009 in Mozambique (UNICEF, 2019). The proportion of never-married 15–19 year-olds who had not used a condom at last sex was 56%, and multiple sexual partners was 35% among boys and 5% among girls in 2009 in Mozambique (Doyle et al., 2012). In 2011, the ‘Mozambique Demographic Health Survey (DHS)’ showed among female adolescents that 8% had used modern contraceptives (UNICEF, 2019). The teenage pregnancy prevalence in Mozambique of youth between 20 and 24 years of age was 40.2% in 2011 (UNFPA, 2013). The HIV prevalence was 6.5% among girls and 1.5% among boys in the 15–19 age group in 2009 in Mozambique (UNICEF, 2019).

Among adolescents in other African countries, in Ghana in 2012, 33.5% ever had sex, 73.8% had not used a condom at last sex, and 32.5% had multiple sexual partners (Kugbey, Ayanore, Amu, Oppong Asante, & Adam, 2018), in Namibia in 2004, 33.2% ever had sex and 17.1% had multiple sexual partners (Chinsembu, Siziya, Muula, & Rudatsikira, 2008), and 25.3% multiple sexual partners in 2013 (Peltzer & Pengpid, 2017), and in Uganda in 2003, 14.9% of boys and 7.9% of girls were sexually active in the past year, 22.7% had not used a condom at last sex, and 60.9% had multiple sexual partners (Twa-Twa, Oketcho, Siziya, & Muula, 2008). In a community survey among adolescents (15–19 years) in Uganda, Tanzania, Nigeria, Ghana, Eswatini, Ethiopia and Burkina Faso between 2015 and 2017, 25.9% ever had sex, and among sexually active early sexual debut (<15 years) was 21% among girls and 28% among boys, unprotected last sex was 46% among girls and 40% among boys, and 37% of girls reported to have been pregnant and 8% of boys had made someone pregnant (Berhane et al., 2020).

In ‘15 year-old school children across 30 countries in Europe, Israel and Canada, 27% had had sexual intercourse and 14% had not used the contraceptive pill or condoms at last sex’ (Nic Gabhainn, Baban, Boyce, & Godeau, 2009), and in a study among 15 year-olds in 10 European countries, the prevalence of sexual initiation was 18.8%, and among sexually active, 52.4% had >1 sexual partner, 14.7% did not or rarely used condoms and 3.0% were involved in pregnancy (Gambadauro, Carli, Wasserman, et al., 2018).

Although the prevalence figures of sexual behaviour may differ in different parts of the world, the associations between sexual and non-sexual risk behaviours, as well as the role of psychosocial modulators, may follow similar patterns across countries and cultures. As reviewed in Peltzer and Pengpid (2016, p. 406), ‘factors associated with sexual risk behaviour among adolescents (ever had sex, early sexual debut, no condom use, and no contraceptive use), include, male sex, older age, substance use, psychological distress, school truancy, lack of parental and peer support’.

We have insuffient recent national data on sexual behaviour and its risk factors among school adolescents in Mozambique. Knowing the occurrence and factors associated with sexual behaviour and its risk factors among adolescents in Mozambique will help in informing intervention strategies targeting the delay of sexual initiation and promoting ‘safer sex’. Therefore, this secondary analysis aimed to estimate the prevalence and predictors of sexual risk behaviours among school-adolescents in Mozambique in 2015.

## Methods

### Sample and procedure

This study used secondary data analysis of a national sample of school adolescents in the 2015 Mozambique cross-sectional GSHS (WHO, 2019). The Mozambique GSHS dataset is publicly available for download at https://www.who.int/ncds/surveillance/gshs/mozambiquedataset/en/ ‘A two-stage cluster sample design was used to produce data representative of all Grade 8–12 students in Mozambique. At the first stage, schools were selected with probability proportional to enrolment size. At the second stage, classes were randomly selected and all students in selected classes were eligible to participate, and responded to a self-administered questionnaire after written consent was obtained’ (WHO, 2019). The World Health Organization and an ethics committee in Mozambique provided ethics approval (WHO, 2019).

## Measures

The questionnaire use in shown in Table 1 (WHO, 2019). Sexual risk behaviour was assessed with questions on ever having had sexual intercourse, age of sexual debut, number of people having had sexual intercourse with in a lifetime, condom use at last sexual intercourse, and birth control use at last sexual intercourse. Individual sexual risk behaviours were defined as ever having had sex, early sexual debut (<14 years), having had two or more sexual partners in a lifetime, non-condom use at last sex and non-birth control use at last sex. A composite sexual risk behaviour measure included having had sex, early sexual debut (<14 years), having had two or more sexual partners in a lifetime, and non-condom use at last sex; non-birth control use was excluded due to overlap with non-condom use at last sex, following previous studies (Carver, Dévieux, Gaston, Altice, & Niccolai, 2014). ‘The psychological distress items (no close friends, loneliness, anxiety, suicidal ideation and suicide attempt) were summed, and grouped into 0 = 0, 1 = 1 single and 2–5 = 2 multiple’ (Pengpid & Peltzer, 2019, p. 409). ‘The four items on parental or guardian support were summed, and classified into three groups, 0–1 low, 2 medium and 3–4 high support’ (Pengpid & Peltzer, 2019, p. 409).

**Table 1.** Questionnaire items.

Indicator	Item	Responses (coding scheme)
Sex	‘What is your sex?’	‘Male, Female’
Age	‘How old are you?’	‘11 years old or younger to 18 years old or older’
Hunger (proxy for socioeconomic status)	‘During the past 30 days, how often did you go hungry because there was not enough food in your home?’	‘1 = never to 5 = always (coded 1–3 = 0 and 4–5 = 1)’
Sexual behaviour		
Ever sex	‘Have you ever had sexual intercourse?’	‘Yes, No’ (coded yes=1, no=0)
Age of sexual initiation	‘How old were you when you had sexual intercourse for the first time?’	‘I have never had sexual intercourse 11 years old or younger to 18 years old or older’
Number of sex partners	‘During your life, with how many people have you had sexual intercourse?’	‘I have never had sexual intercourse, 1 person to 6 or more people’
Condom use	‘The last time you had sexual intercourse, did you or your partner use a condom?’	‘I have never had sexual intercourse, Yes, No, I do not know’
Birth control use	‘The last time you had sexual intercourse, did you or your partner use any method of birth control, such as withdrawal, rhythm (safe time), birth control pills, or any other method to prevent pregnancy?’	‘I have never had sexual intercourse, Yes, No, I do not know’
Substance use		
Current tobacco use	‘During the past 30 days, on how many days did you smoke cigarettes/use any tobacco products other than cigarettes, such as such as *suruma*?’	‘1 = 0 days to 7 = All 30 days (coded 1 = 0 and 2–7 = 1)’
Current alcohol use	‘During the past 30 days, on how many days did you have at least one drink containing alcohol?’	‘1 = 0 days to 7 = All 30 days (coded 1 = 0, 2–7 = 1)’
Cannabis use	‘During your life, how many times have you used marijuana (also called *passa)*?’	‘1 = 0 times to 5 = 20 or more times (coded 1 = 0 and 2–5 = 1)’
Amphetamine use	‘During your life, how many times have you used amphetamines or methamphetamine (also called *drogas injectaveis*)?’	‘1 = 0 times to 5 = 20 or more times (coded 1 = 0 and 2–5 = 1)’
Psychological distress		
No close friends	‘How many close friends do you have?’	‘1 = 0–4 = 3 or more (coded 1+=0, 0 = 1)’
Loneliness	‘During the past 12 months, how often have you felt lonely?’	‘1=never to 5=always (coded 1–3 = 0 and 4–5 = 1)’
Anxiety	‘During the past 12 months, how often have you been so worried about something that you could not sleep at night?’	‘1=never to 5=always (coded 1–3 = 0 and 4–5 = 1)’
Suicide ideation	‘During the past 12 months, did you ever seriously consider attempting suicide?’	‘Yes, No’
Suicide attempt	‘During the past 12 months, how many times did you actually attempt suicide?’	‘1 = 0 times to 5 = 6 or more times (coded 1 = 0 and 2–5 = 1)’
Protective factors		
School attendance	‘During the past 30 days, on how many days did you miss classes or school without permission?’	‘1 = 0 days to 10 or more days (coded 1 = 1)’
Peer support	‘During the past 30 days, how often were most of the students in your school kind and helpful?’	‘1=never to 5=always (coded 1–3 = 0 and 4–5 = 1)’
Parental supervision	‘During the past 30 days, how often did your parents or guardians check to see if your homework was done?’	‘1=never to 5=always (coded 1–3 = 0 and 4–5 = 1)’
Parental connectedness	‘During the past 30 days, how often did your parents or guardians understand your problems and worries?’	‘1=never to 5=always (coded 1–3 = 0 and 4–5 = 1)’
Parental bonding	‘During the past 30 days, how often did your parents or guardians really know what you were doing with your free time?’	‘1=never to 5=always (coded 1–3 = 0 and 4–5 = 1)’
Parental respect for privacy	‘During the past 30 days, how often did your parents or guardians go through your things without your approval?’	‘1=never to 5=always (coded 1–3 = 0 and 4–5 = 1)’

## Data analysis

Data analysis was performed using STATA software version 15.0 (Stata Corporation, College Station, TX, USA). Descriptive statistics was used to describe the sample. Sex differences in the proportion of variables were calculated with Pearson Chi-square statistics. Logistic regression was used on the whole sample to identify predictors of individual sexual risk behaviours (non-birth control use at last sex non-condom use at last sex, multiple sexual partners, early sexual debut, and ever had sex) and a composite measure of multiple sexual risk behaviour (at least two risk behaviours had to be present). Co-variates were included based on a previous review (Peltzer & Pengpid, 2016). Taylor linearisation procedures were utilised in all statistical operations in order to account for the sampling weight and the multi-stage design of the study. Missing cases were not included in the analysis. The level of significance was set at *p *< 0.05.

## Results

### Characteristics of the sample and sexual behaviour

The overall (school and individual) study response rate was 80% (WHO, 2019). The sample consisted of 1918 in-going adolescents from Mozambique, with a median age of 15 years (IQR: 15–18). More than half (57.4%) of the students ever had sex, 68.4% among boys and 45.8% among girls. Among students that had been sexually active, 41.5% had early sexual debut (<14 years), 57.9% had multiple sexual partners, 42.0% had not used birth control at last sex, 25.0% had not used a condom at last sex, and 63.7% had engaged in multiple sexual risk behaviours. The proportion of having two of more sexual risk behaviours, multiple sex partners, sexual initiation, and early sexual debut was higher in boys than in girls (see Table 2).

**Table 2.** Sample and sexual behaviour characteristics among adolescents in Mozambique, 2015.

Study variable	All	Males	Females	*P*-value
	*N* (%)	*N* (%)	*N* (%)	
Sociodemographic variables				
All	1918			
Age in years				
14 or less	357 (29.4)	172 (25.0)	180 (32.9)	0.050
15–16	691 (32.8)	357 (39.8)	342 (36.2)	
17 or more	784 (37.8)	457 (35.2)	340 (30.9)	
Hunger (mostly/always)	195 (11.3)	107 (12.0)	86 (10.5)	0.518
Sexual behaviour				
Ever sex	993 (57.4)	599 (68.4)	369 (45.8)	<0.001
Early sexual debut (<14 years)^a^	284 (41.5)	227 (47.5)	51 (29.0)	<0.001
Multiple sexual partners^a^	486 (57.9)	361 (64.5)	114 (43.8)	<0.001
No condom use at last sex^a^	197 (25.0)	140 (26.7)	54 (22.1)	0.357
No birth control use at last sex^a^	282 (42.0)	188 (44.0)	90 (38.5)	0.316
Multiple sexual risk behaviour^a^	544 (59.4)	401 (68.4)	133 (36.5)	<0.001
* Substance use*				
Current tobacco use	83 (5.5)	52 (5.8)	31 (5.2)	0.667
Days drinking alcohol				
0	1538 (88.4)	798 (87.5)	693 (88.5)	0.554
1 or 2	171 (8.3)	88 (8.5)	82 (8.7)	
3–30	66 (3.3)	43 (3.9)	23 (2.8)	
Ever cannabis and/or amphetamine use	35 (1.7)	25 (2.2)	9 (1.2)	0.183
Psychological distress				
0	965 (58.0)	520 (62.1)	427 (54.5)	0.062
1	452 (25.9)	234 (25.4)	208 (26.3)	
2–5	269 (16.1)	121 (12.5)	135 (19.2)	
* Protective factors*				
School attendance	1384 (75.4)	706 (74.5)	641 (76.8)	0.291
Peer support	620 (33.3)	330 (33.7)	284 (32.7)	0.765
Parental support				
Low	668 (34.8)	361 (37.0)	307 (31.5)	0.362
Medium	450 (26.1)	238 (25.6)	208 (26.4)	
High	608 (39.0)	311 (37.4)	300 (42.0)	

### Associations with sexual risk behaviour

In adjusted logistic regression analysis, male sex was associated with ever sex (AOR: 2.20, 95% CI: 1.54–3.14), early sexual debut (AOR: 4.48, 95% CI: 2.51–7.98), multiple sexual partners (AOR: 4.44, 95% CI: 3.16–6.25), non-condom use at last sex (AOR: 1.96, 95% CI: 1.08–3.53), non-birth control use at last sex (AOR: 2.28, 95% CI: 1.45–3.57), and multiple sexual risk behaviour (AOR: 4.53, 95% CI: 3.07–6.70). Compared to participants aged 14 years or less, participants 17 years or older were more likely to ever had sex (AOR: 4.46, 95% CI: 2.51–7.93), had multiple sexual partners (AOR: 6.53, 95% CI: 3.33–12.85), had not used a condom at last sex (AOR: 2.23, 95% CI: 1.07–4.66), had not used birth control at last sex (AOR: 3.14, 95% CI: 1.72–5.72), and had multiple sexual risk behaviours (AOR: 3.25, 95% CI: 1.75–6.04). Students who had reported that they were mostly or always hungry were not significantly more likely engaging in any of the sexual risk indicators than students who said that they were never, rarely or sometimes hungry. Current alcohol use increased the odds for all five sexual risk behaviours (ever had sex = AOR: 2.88, 95% CI: 1.39–5.98; early sexual debut = AOR: 1.37, 95% CI: 1.21–4.66; multiple sexual partners = AOR: 2.11, 95% CI: 1.34–3.32; non-condom use at last sex = AOR: 1.63, 95% CI: 1.01–2.64; non-birth control use at last sex = AOR: 1.92, 95% CI: 1.21–3.04) and multiple sexual risk behaviour (AOR: 2.25, 95% CI: 1.20–4.23), while ever cannabis and/or amphetamine use increased the odds for early sexual debut (AOR: 2.83, 95% CI: 1.26–6.38). Psychological distress was not not significantly associated with any of the sexual risk indicators. School attendance was protective from multiple sexual partners (AOR: 0.64, 05% CI: 0.43–0.95) and multiple sexual risk behaviour (AOR: 0.65, 95% CI: 0.45–0.98). High parental support was protective against non-condom use (AOR: 0.44, 95% CI: 0.22–0.89) and non-birth control use at last sex (AOR: 0.57, 95% CI: 0.38–0.87) but not against the other sexual risk behaviours lack of peer support was not associated with any sexual risk behaviour (see Tables 3 and 4).

**Table 3.** Associations with ever had sex, early sexual debut and multiple sexual partners.

Variable	Ever had sex (*N* = 1168)	Early sexual debut (*N* = 1201)	Multiple sexual partners (*N* = 1232)
	AOR (95% CI)	AOR (95% CI)	AOR (95% CI)
Sociodemographics			
Sex			
Female	1 (Reference)	1 (Reference)	1 (Reference)
Male	2.20 (1.54, 3.14)***	4.48 (2.51, 7.98)***	4.44 (3.16, 6.25)***
Age in years			
14 or less	1 (Reference)	1 (Reference)	1 (Reference)
15–16	1.97 (1.19, 3.26)**	1.06 (0.60, 1.87)	3.16 (1.83, 5.45)***
17 or more	4.46 (2.51, 7.93)***	0.71 (0.35, 1.46)	6.53 (3.33, 12.85)***
Experience hunger	0.85 (0.49, 1.48)	0.96 (0.45, 2.06)	0.96 (0.49, 1.89)
Substance use			
Current tobacco use	3.46 (0.82, 14.65)	1.11 (0.33, 3.80)	1.00 (0.38, 2.64)
Current alcohol use	2.88 (1.39, 5.98)**	1.37 (1.21, 4.66)*	2.11 (1.34, 3.32)***
Ever cannabis and/or amphetamine use	5.84 (0.80, 42.39)	2.83 (1.26, 6.38)*	1.23 (0.42, 3.61)
Psychological distress			
0	1 (Reference)	1 (Reference)	1 (Reference)
1	1.33 (0.98, 1.79)	1.20 (0.81, 1.79)	0.78 (0.52, 1.17)
2–5	1.66 (0.86, 3.20)	1.34 (0.67, 2.71)	0.86 (0.50, 1.50)
Protective factors			
School attendance	0.72 (0.52, 1.00)	0.68 (0.45, 1.04)	0.64 (0.43, 0.95)*
Peer support	0.75 (0.51, 1.09)	1.01 (0.58, 1.77)	1.05 (0.72, 1.54)
Parental support			
Low	1 (Reference)	1 (Reference)	1 (Reference)
Medium	1.06 (0.74, 1.53)	1.16 (0.70, 1.93)	1.02 (0.70, 149)
High	0.93 (0.71, 1.22)	0.89 (0.58, 1.39)	1.01 (0.64, 1.58)

**Table 4.** Associations with non-condom use and non-birth control use at last sex and multiple sexual risk behaviours.

Variable	Non-condom use (*N* = 1219)	Non-birth control use (*N* = 1143)	Multiple sexual risk behaviours (*N* = 1118)
	AOR (95% CI)	AOR (95% CI)	AOR (95% CI)
Sociodemographics			
Sex			
Female	1 (Reference)	1 (Reference)	1 (Reference)
Male	1.96 (1.08, 3.53)*	2.28 (1.45, 3.57)***	4.53 (3.07, 6.70)***
Age in years			
14 or less	1 (Reference)	1 (Reference)	1 (Reference)
15–16	1.50 (0.74, 3.05)	1.78 (1.00, 3.17)	1.98 (1.19, 3.29)***
17 or more	2.23 (1.07, 4.66)*	3.14 (1.72, 5.72)***	3.25 (1.75, 6.04)***
Experience hunger	0.60 (0.27, 1.31)	0.83 (0.52, 1.34)	1.01 (0.51, 1.99)
Substance use			
Current tobacco use	1.50 (0.50, 4.45)	2.54 (0.95, 6.81)	1.28 (0.39, 4.17)
Current alcohol use	1.63 (1.01, 2,64)*	1.92 (1.21, 3.04)**	2.25 (1.20, 4.23)*
Ever cannabis and/or amphetamine use	0.72 (0.29, 1.75)	1.05 (0.20, 5.49)	1.91 (0.93, 3.94)
Psychological distress			
0	1 (Reference)	1 (Reference)	1 (Reference)
1	1.12 (0.69, 1.81)	0.76 (0.48, 1.19)	1.00 (0.70, 1.43)
2–5	0.91 (0.49, 1.69)	1.11 (0.59, 2.09)	0.99 (0.52, 1.88)
Protective factors			
School attendance	0.97 (0.61, 1.52)	0.87 (0.54, 1.41)	0.65 (0.45, 0.98)*
Peer support	1.09 (0.58, 2.03)	0.90 (0.63, 1.29)	0.99 (0.64, 1.53)
Parental support			
Low	1 (Reference)	1 (Reference)	1 (Reference)
Medium	0.78 (0.40, 1.53)	0.98 (0.68, 1.41)	1.08 (0.66, 1.76)
High	0.44 (0.22, 0.89)*	0.57 (0.38, 0.87)**	0.82 (0.57, 1.18)

## Discussion

The study found a high prevalence of sexual initiation (57.8%) among school-going adolescents in Mozambique, which was higher than in Ghana (33.5%) (Kugbey et al., 2018), in Namibia 33.2% (Chinsembu et al., 2008), Uganda (11.4%) (Twa-Twa et al., 2008), in a community survey in seven African countries (25.9%) (Berhane et al., 2020), in 30 countries in Europe, Israel and Canada (27%) (Nic Gabhainn et al., 2009), and in a 10 European countries study (18.8%) (Gambadauro, Carli, Wasserman, et al., 2018). Among the sexually active, 25.0% had not used a condom at last sex, which is much lower (56%) than in a 2009 survey among adolescents in Mozambique (Doyle et al., 2012), among adolescents in Ghana (73.8%) (Kugbey et al., 2018), and in seven African countries (46% among girls and 49% among boys) (Berhane et al., 2020), but similar to adolescents in Uganda (22.7%) (Twa-Twa et al., 2008). In addition, high sexual risk behaviour was found, in terms of early sexual debut (<15 years) (41.5%), multiple sex partners (57.9%), non-birth control use (last sex) (42.0%), and engagement in two or more sexual risk behaviours (59.4%). These results seem to be higher than in previous surveys among adolescents, such as early sexual debut (19.5%) in Mozambique (UNICEF, 2019) and in seven African countries (<15 years, 21% among girls and 28% among boys) (Berhane et al., 2020), multiple sexual partners (20%) in Mozambique (Doyle et al., 2012), but lower in non-contraceptive use (88.5%) in Mozambique (UNICEF, 2019) and higher in non-contraceptive use than in Europe, Israel and Canada (14%) (Nic Gabhainn et al., 2009). So, it appears that protective sexual intercourse increased but also early sexual debut and multiple sexual partners increased among adolescents in Mozambique.

Male sex and/or increasing age was associated with multiple sexual risk behaviour, non-birth control use (last sex), non-condom use (last sex), multiple sexual partners, early sexual debut, and ever had sex. Similar results were shown in previous investigations (Carver et al., 2014; Chinsembu et al., 2008; Kugbey et al., 2018; Mmari & Blum, 2009; Peltzer & Pengpid, 2016; Twa-Twa et al., 2008), which may support the case of sexual risk intervention programmes targeting male adolescents at an earlier age than their female counterpart. Unlike in former research (Sanchez et al., 2013), this study showed a non-association between frequent hunger experience (or lower socioeconomic status) sexual risk behaviours. A possible explanation for this finding is that the prevalence of mostly or always experiencing hunger was low and the concept of socioeconomic status is assessed more comprehensively in other studies, such as including education of the household head and a list of household possessions (Sanchez et al., 2013).

In agreement with a number of previous studies (Carver et al., 2014; Chinsembu et al., 2008; Kugbey et al., 2018; Mmari & Blum, 2009; Page & Hall, 2009; Peltzer & Pengpid, 2016; Siziya, Muula, Kazembe, & Rudatsikira, 2008), this survey showed that drug and alcohol use increased the likelihood of engaging in sexual risk behaviours, calling for reproductive and sexual health programmes to integrate alcohol and drug use (Page & Hall, 2009; Peltzer & Pengpid, 2016). Unlike in previous investigations (Kugbey et al., 2018; Page & Hall, 2009; Peltzer, 2010; Peltzer & Pengpid, 2016), psychological distress was in this study not associated with single and multiple sexual risk behaviours. A possible explanation for the non-significant association between psychological distress and sexual risk behaviours may be related to how psychological distress was measured, namely in this study with internalising symptoms (loneliness, no close friends, suicidal ideation and attempt), while in a study among European adolescents externalising symptoms, such as hyperactivity and conduct, was positively associated with sexual initiation (Gambadauro, Carli, Hadlaczky, et al., 2018). In addition, the strength of the associations between sexual risk taking and psychological problems variates depending on factors such as the severity of the symptoms and the sex of the pupil (Gambadauro, Carli, Hadlaczky, et al., 2018).

School attendance was found to be protective against having multiple sexual risk behaviours and multiple sexual partners, which is consistent with previous research findings (Mmari & Blum, 2009; Peltzer & Pengpid, 2016). Interventions preventing school truancy and promoting school attendance may also be beneficial in sexual risk behaviour reduction. Partially in agreement with former research studies (Carver et al., 2014; Mmari & Blum, 2009; Peltzer & Pengpid, 2011), this survey showed that parental support was protective against non-birth control and non-condom use. There is an increased need for comprehensive sexual and reproductive health education in secondary school and peer-led programmes in Mozambique (Miedema & Oduro, 2017).

## Limitations of the study

The GSHS only includes adolescents that attend school, excluding out-off school youth. Adolescents who have dropped out of school may be more vulnerable to sexual risk behaviour. The GSHS Mozambique was cross-sectional by design, which precludes causative inferences between study variables. Further, the self-reported data collection may have led to biased responses, in particular regarding sensitive issues, such as sexual behaviour. The GSHS does not provide a definition of ‘sexual intercourse’, and therefore it is possible that some students misinterpreted the meaning, but the same question is used in various other surveys among adolescents (e.g. Gambadauro, Carli, Wasserman, et al., 2018).

## Conclusion

More than half of the students ever had sex, had multiple sexual partners, and multiple sexual risk behaviours. Sexual risk behaviours were higher in students who engaged in substance use, were older, were male and were absent from school. Taking the identified factors associated with sexual risk behaviours into account, will be important in the design and scaling up of sexuality and reproductive health education among school adolescents in Mozambique.

## Footnotes

^a^
Of sexually active.
